# Development of Polymeric Nanocomposite (Xyloglucan-co-Methacrylic Acid/Hydroxyapatite/SiO_2_) Scaffold for Bone Tissue Engineering Applications—In-Vitro Antibacterial, Cytotoxicity and Cell Culture Evaluation

**DOI:** 10.3390/polym12061238

**Published:** 2020-05-29

**Authors:** Muhammad Umar Aslam Khan, Hassan Mehboob, Saiful Izwan Abd Razak, Mohd Yazid Yahya, Abdul Halim Mohd Yusof, Muhammad Hanif Ramlee, T. Joseph Sahaya Anand, Rozita Hassan, Athar Aziz, Rashid Amin

**Affiliations:** 1 School of Biomedical Engineering, Med-X Research Institute, Shanghai Jiao Tong University (SJTU), 1954 Huashan Road, Shanghai 200030, China; umar-786@sjtu.edu.cn; 2 School of Biomedical Engineering and Health Sciences, Universiti Teknologi Malaysia, Skudai 81300, Johor, Malaysia; saifulizwan@utm.my; 3 Department of Engineering Management, College of Engineering, Prince Sultan University, P.O. Box No. 66833, Rafha Street, Riyadh 11586, Saudi Arabia; hmehboob@psu.edu.sa; 4 Centre for Advanced Composite Materials, Universiti Teknologi Malaysia, Skudai 81300, Johor, Malaysia; yazidyahya@utm.my; 5 School of Chemical and Energy Engineering, Faculty of Engineering, Universiti Teknologi Malaysia, Skudai 81300, Johor, Malaysia; halimy@cheme.utm.my; 6 Medical Devices and Technology Centre (MEDiTEC), Institute of Human-Centered Engineering (iHumEn), Universiti Teknologi Malaysia, Skudai 81300, Johor, Malaysia; m.hanif@utm.my; 7 Sustainable and Responsive Manufacturing Group, Fakulti Kejuruteraan Pembuatan, Universiti Teknikal Malaysia Melaka, Hang Tuah Jaya, Durian Tunggal 76100, Melaka, Malaysia; anand@utem.edu.my; 8 Orthodontic Unit, School of Dental Science, Universiti Sains Malaysia, Kelantan 16150, Malaysia; rozitakb@usm.my; 9 School of Environment and Life Sciences, Biomedical Research Centre University of Salford, Manchester M5 4WT, UK; 10 Department of Biology, College of Sciences, University of Hafr Al Batin, Hafar Al-Batin 39524, Saudi Arabia

**Keywords:** antibacterial active, biocompatibility, nanotechnology, nanocomposite scaffolds, bone tissue engineering

## Abstract

Advancement and innovation in bone regeneration, specifically polymeric composite scaffolds, are of high significance for the treatment of bone defects. Xyloglucan (XG) is a polysaccharide biopolymer having a wide variety of regenerative tissue therapeutic applications due to its biocompatibility, in-vitro degradation and cytocompatibility. Current research is focused on the fabrication of polymeric bioactive scaffolds by freeze drying method for nanocomposite materials. The nanocomposite materials have been synthesized from free radical polymerization using n-SiO_2_ and n-HAp XG and Methacrylic acid (MAAc). Functional group analysis, crystallinity and surface morphology were investigated by Fourier transform infrared spectroscopy (FTIR), X-ray diffraction analysis (XRD) and scanning electron microscopy (SEM) techniques, respectively. These bioactive polymeric scaffolds presented interconnected and well-organized porous morphology, controlled precisely by substantial ratios of n-SiO_2_. The swelling analysis was also performed in different media at varying temperatures (27, 37 and 47 °C) and the mechanical behavior of the dried scaffolds is also investigated. Antibacterial activities of these scaffolds were conducted against pathogenic gram-positive and gram-negative bacteria. Besides, the biological behavior of these scaffolds was evaluated by the Neutral Red dye assay against the *MC3T3-E1* cell line. The scaffolds showed interesting properties for bone tissue engineering, including porosity with substantial mechanical strength, biodegradability, biocompatibility and cytocompatibility behavior. The reported polymeric bioactive scaffolds can be aspirant biomaterials for bone tissue engineering to regenerate defecated bone.

## 1. Introduction

Bone tissue damage caused by trauma, injury or disease needs progressive approaches for tissue repair and development. Bone tissue engineering is an innovative technique for the treatment of defected and broken bones using composite scaffolds. Bone is a vital part of the body, supporting and protecting the soft tissues and help to maintain the structure. In recent years, scientists have contributed a great deal of effort for bone tissue engineering and regenerative medicine to resolve bone augmentation complications [1,2]. Numbers of bone grafting have increased significantly in the last few decades due to the increasing number of bone damage in accidents or other traumas. Biomaterial-based bone regeneration is an effective alternative because of the materials’ intrinsic properties. The bioactive composite scaffolds are implanted to treat defected or fractured bone sites and integrated into the osteochondral system [3,4]. The limitations of auto-grafts because of donor availability issues compelled scientists to develop biocompatible composite materials for bone tissue engineering. We are reporting here polymeric scaffolds, synthesized for the bone tissue applications, because of their anticipated porous, physicochemical, degradability and biomechanical properties with tunable characteristics [5,6]. Typically, tissue-engineering approaches involve porous composite scaffolds for living cells. The ceramic material offers sufficiently porous, rough morphology and polymer imitation of the extracellular matrix. Due to biocompatible, biodegradable and bioactive activities, the polysaccharides have drawn the attention of researchers in tissue engineering. Because of its physical and chemical, it proved to be a potential candidate for wound dressing and tissue engineering because of non-allergic or non-inflammatory response [7,8].

Xyloglucan (XG) is also a well-known biological macromolecule with wide biomedical applications. It is famous for pharmaceutical applications due to controlled swelling ability, biocompatibility and biodegradable properties with water-soluble and non-toxic and nonirritant polysaccharide. XG consists of polysaccharide 1,4-β-D-glucan backbone with partial substitutions of the 1,6-β-D-xylopyranosyl side chain and additionally substituted with 1,2-β-D-galactopyranosyl residue [9,10]. The commercial tamarind kernel powder is a significant source of XG raw material. XG has bared many applications; it has been used as a common excipient in cosmetics, food additive and acts as stabilizer and thickener agents [11,12]. A suitable polymeric composite was prepared via blending with cations Ca^2+^ and these cations interacted with a negatively charged hydroxyl group (OH^−^) of polysaccharide chain due to the electrostatic force of interaction, which resulted in the formation of the 3D network. Polysaccharide-based scaffolds have a limitation because of poor/inadequate mechanical properties. Ceramic-based materials (hydroxyapatite (HAp)) have substantial mechanical and biocompatibility for hard tissue engineering [13]. In the present study, silica has been selected as an essential material, well known for its lightweight and excellent mechanical properties. Biomaterials based on nanocomposites gained the attention of scientists because of their porous, wide surface area and biocompatibility, compared to other ceramic materials [14,15]. Silicon dioxide or silica (SiO_2_) demonstrates numerous applications in drug delivery and tissue engineering as an ultimate biomaterial. SiO_2_ nanoparticles have a wide surface area, resembling the biopolymer matrix of bio-composite materials. Bioglass with silica nanoparticles not only provides mineralizing, porous and lightweight capabilities for polymeric scaffolds but also increases its strength as a polymer composite scaffold, having desirable biomaterial properties [16,17].

Nisbet et al. has used XG and functionalized it by positive charge molecules of poly(D-lysine), which showed an appropriate atmosphere for cell culture, migration and proliferation. Moreover, the thermo-responsive hydrogels have shown returns of spinal cord injuries treatments [18]. Shaw et al. prepared carboxymethyl tamarind gum-based films for skin tissue engineering by the phase-separation method. They found that enhanced proliferation of human keratinocytes than the control group. It was found that drug-loaded films showed good antimicrobial behavior against Escherichia coli and results showed that films are suitable as matrices for skin tissue engineering [19]. Yoganand and coworkers have synthesized glass-ceramics from natural bovine HAp/SiO_2_–CaO–MgO glass composites by the brisk process. They have performed biological studies on cultures of human fibroblast cells and found promoted adherence and growth [20]. K. Mediaswanti et al. have deposited SiO_2_/HAp onto a titanium and tantalum surface by electron beam evaporation and magnetron sputtering. They found that HAp/SiO_2_ coated surface was less desirable results of bacteria adherence than non-coated surface [21].

**Figure 1:**
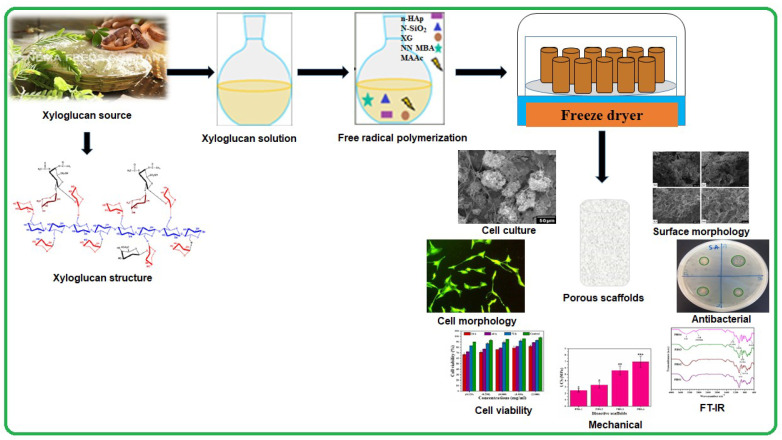
The schematic diagram describes the fabrication, characterizations, an in vitro study of polymeric bioactive scaffolds using nanoparticles of ceramic doped in a grafted biopolymeric matrix of xyloglucan.

Hence, considering the physicochemical and biomechanical behavior of biomaterials, the objectives of this article are to prepare, characterize and biological analysis of polymeric bioactive scaffolds (PBSs). Nanoparticles of SiO_2_ (n-SiO_2_, auxiliary component) and nanoparticles of HAp (n-HAp, supplementary constituent) lodged into a grafted biopolymeric network during free-radical polymerization of XG with methacrylic acid (MAAc). According to our best knowledge, the methodology of polymeric bioactive scaffolds has never reported up till now. n-SiO_2_ doped polymeric bioactive scaffolds have a porous, large surface and biocompatible properties for osteogenesis. The structure and morphology of polymeric bioactive scaffolds were analyzed by Fourier transform infrared spectroscopy (FTIR), X-ray diffraction analysis (XRD), energy-dispersive X-ray spectroscopy (EDS), scanning electron microscopy (SEM) and Brunauer–Emmett–Teller (BET). The mechanical behavior and swelling properties were analyzed using the universal testing machine (UTM) in water and phosphate buffer saline (PBS) solution, respectively. MC3T3-E1 cell line was employed to study the biological behavior of polymeric bioactive scaffolds in vitro. The results of our study revealed, bioactive scaffolds as potential biomaterials to regenerate and repair defective bone in tissue engineering. Figure 1 illustrates the schematic diagram of the reported studies.

## 2. Materials and Methods

### 2.1. Materials

XG was purchased from DSP Gokyo Food and Chemical Co. Ltd., Tokyo, Japan. MAAc and *N*,*N*’-methylene-bis-acrylamide (*N*, *N*-MBA) as a crosslinker, n-HAp (<100 nm particle size) and n-SiO_2_ (10–20 nm particle size) in powder forms were purchased from Sigma-Aldrich, Selangor, Malaysia. Analytical graded Na_2_HPO_4_, NaCl, KCl, K_2_HPO_4_ and HCl were obtained from Merck Darmstadt, Germany for the preparation of buffer saline solution. All chemicals were used without any purification.

### 2.2. Polymeric Bioactive Scaffolds Synthesis

Synthesis of nanocomposite to fabricate polymeric bioactive scaffolds, 2 g XG in deionized-water and shifted in round bottom two-neck flask. The reaction media temperature was held at 60 °C with constant stirring under N_2_ atmosphere and 0,05 g potassium persulfate was used as an initiator. After 20 min, 0.40 mL MAAc and *N*, *N*-MBA (0.05% of MAAc) were poured into the reaction media. After 45 min, different quantities of n-SiO_2_ (0.25, 0.50, 0.75 and 1.0 g) and coded as PBS1, PBS2, PBS3 and PBS4, respectively. Then, n-Hap (2 g) powders were added gradually with continuous stirring for 3 h. Hence, MAAc was grafted into XG using a free-radical polymerization method. As a result, n-SiO_2_ and n-HAp were incorporated into the XG-graft-MAAc polymeric network. On completion, the reaction was halted and the gas flow was removed to cool the reaction media. The residual suspension was separated by vacuum filtration from the reactive mixture. The residues were washed with excessive deionized water, then dried overnight in the oven at 55 °C to obtain the nanocomposite powder. Then the composite powder was prepared with different compositions of n-SiO_2_, as summarized in Table 1. The polymeric nanocomposite powder (0.5 g) was homogeneously mixed in deionized water to form a uniform paste, filled in cylindrical molds (1.5 mm × 6 mm) and kept at −80 °C for 24 h. The cylindrical polymeric bioactive scaffolds were obtained through the freeze-drying method with no significant volume reduction or deformation. The porous morphology of the polymeric bioactive scaffolds was observed through Brunauer–Emmett–Teller (Micromeritics Gemini II 2370, Micromeritics, Norcross, GA, USA).

## 3. Characterization

### 3.1. Fourier Transform Infrared Spectroscopy

Functional groups of the polymeric bioactive scaffolds were investigated through IR Prestige-21, Shimadzu (Kyoto, Japan) with a frequency range of 4000−400 cm^−1^ and resolution of 4.0 cm^−1^ with 200 scans average per spectrum.

### 3.2. Scanning Electron Microscope/Energy Dispersive Spectroscopy

Morphologies of the polymeric bioactive scaffolds have been analyzed by scanning electron microscope (SEM, JEOL-JSM-6480, Akishima, Japan) coupled with energy dispersive spectroscopy (EDS) for elemental quantitative analysis. Cross-sectional pieces of the scaffolds were sliced and placed on a stub and located into the vacuum chamber to collect images at different magnifications.

### 3.3. Mechanical Testing

A universal testing machine (UTM), (Testometrics, UK) was employed for mechanical testing with a loading rate of 5 mm/min. Ultimate compression strength (UCS) of the samples was determined using triplicate.

### 3.4. Swelling Test

Dried specimens of scaffolds were weighted (*W_D_*) at pH 7.4 and 27, 37 and 47 °C, swelling behavior of dried specimens of scaffolds was conducted in deionized H_2_O and PBS solution. PBS solution was prepared in the laboratory by standard method. The pH of the solution was maintained at 7.4 by adding hydrochloric acid (0.1 mol L^−1^) dropwise and raised the volume of PBS solution to 1 L by adding deionized water. Then, scaffolds specimens were soaked and liquid-immersed into pores until equilibrium reached. The swelled specimens of scaffolds took out from liquid and removed the excess surface water to determine their weights (*W_S_*). The swelling (%) of the scaffolds was evaluated using Equation (1).

(1) $$Swelling\;\left(\%\right)=\frac{W_{S}-W_{D}}{W_{D}}\times 100$$

Whereas, *W_S_* = scaffold weight, *W_D_* = Weight of dry scaffold

### 3.5. In Vitro Studies

In vitro studies were conducted from the extracts of polymeric bioactive scaffolds to investigate antibacterial activities and biological activities against various bacterium (*E. coli, S. aureus and* *P. Aeruginosa*) and mouse pre-osteoblast (*MC3T3-E1*) cell lines.

#### 3.5.1. Antibacterial Activities

The antibacterial activities of all samples were studied by the disc diffusion method against gram-negative and gram-positive model bacterial strains (*E. coli, S. aureus and* *P. Aeruginosa*). These bacterial strains were incubated at 37 °C to study antibacterial activities of all polymeric bioactive scaffolds. Hot molten (15 mL) of agar was poured into three sterile Petri-plates and left them for solidification. After that, these bacterial cultures were spread uniformly using sterile cotton swab over solidified agar [22]. 85 μL of each scaffold extract was put over bacterial cultured plates and incubated into the oven for 24 h at 37 °C.

#### 3.5.2. Extract of Scaffold Preparation

All polymeric bioactive scaffolds exhibited characteristics like porosity, swelling and mechanical properties. Five different concentrations (0.125–2.00 mg mL^−1^) were prepared from all samples of polymeric bioactive scaffolds in liquid to evaluate cell viability. The bottoms of 24 well plates were fine coated for all samples in triplicate and sterilized under UV-light for 1 h.

#### 3.5.3. Cell Culture and Morphological Analysis

Mouse pre-osteoblast (*MC3T3-E1*) cell line obtained from ATCC (American Type Culture Collection) was kept in α-MEM (without ascorbic acid Gibco™ A10490-9, New York, NY, USA) with 10% FBS (Fetal Bovine Serum Gibco™ 12662011, New York, NY, USA) and 100 U/mL Penicillin/Streptomycin solution (Gibco™ 15140122, New York, NY, USA). To conduct microscopy-based morphological analysis, all wells of 24 well plates were seeded with approximately 5000 cells per cm^2^ for five different concentrations using the same media in triplicate and incubated at 37 °C for 72 h in 5% CO_2_ with 90% humidity. The Nikon Eclipse TS100 inverted microscope (Nikon Instruments Inc, New York, NY, USA) was employed to observe cell morphology and cell viability assay for 72 h changes.

#### 3.5.4. Cell Cultural and Morphological Studies

Cell culture images of the polymeric bioactive scaffold were captured using SEM (JSM 6940A, Jeol, Tokyo, Japan). At room temperature, the attached cells were washed with PBS solution using absolute ethanol for 7 min. Then, samples were gold-sputtered and analyzed under 1 kV voltage, 7 × 10^−2^ bar pressure and 20 mA deposition current for 2.0 min.

#### 3.5.5. Cell Culture Viability

Mouse pre-osteoblast (*MC3T3-E1*) cell line obtained from ATCC was kept in α-MEM (without ascorbic acid Gibco™ A10490-9, New York, NY, USA) with 10% FBS (Fetal Bovine Serum Gibco™ 12662011, New York, NY, USA) and 100 U/mL Penicillin/Streptomycin solution (Gibco™ 15140122, New York, NY, USA). Microscopic based cell morphology was observed. Approximately 5,000 cells per cm^2^ were seeded into wells of 12 well plates with five different concentrations of PBS3 and incubated at 37 °C for 72 h in 5% CO_2_ with 95% humidity. Gelatin coating is frequently used to enhance surface cell adherence. Herein, gelatin with a concentration of 0.1% was used as a positive control. The cells were seeded with five different concentrations (0.125–2.00 mg mL^−1^) with 1% dimethyl sulfoxide (DMSO) as a negative control and non-treated cells as a positive control. Cells were incubated for 24, 48, 72 h and the Neutral Red uptake assay was performed reported by *Repetto* et al. [23]. Every day, three wells for each concentration and three for negative control were taken out. The treated and control cells were incubated in neutral red medium (40 µgmL^−1^ Neutral Red medium) for 2 h. Then, the incubated cells were washed with an excessive amount of PBS solution to remove the Neutral Red medium and all samples were then transferred to a de-staining solution (1% glacial acetic acid 49% distilled water and 50% ethanol) for 5 min at room temperature. The spectrophotometer was used to measure optical density at 540 nm and cell viability (%) was determined by Equation (2).

(2) $$\mathrm{Cell}\;\mathrm{viability}\;\left(\%\right)=\frac{OD_{S}}{OD_{C}}\times 100$$

where *OD_S_* and *OD_C_* for sample concentration and *OD_C_* are *OD* for positive control having untreated cells.

#### 3.5.6. Statistical Analysis

Experimental data was conducted in triplicate form and presented with mean standard errors (S.E). The statistical analysis was calculated using statistical analysis system software (IBM, SPSS Statistics 21). The means and standard errors of means (mean ± S.E) were calculated for every analysis and S.E values have displayed as Y-error bars in Figures. The error bars displayed standard deviations (*p* < 0.05 (5 %); size of the sample n = 3).

## 4. Results and Discussion

**Figure 2:**
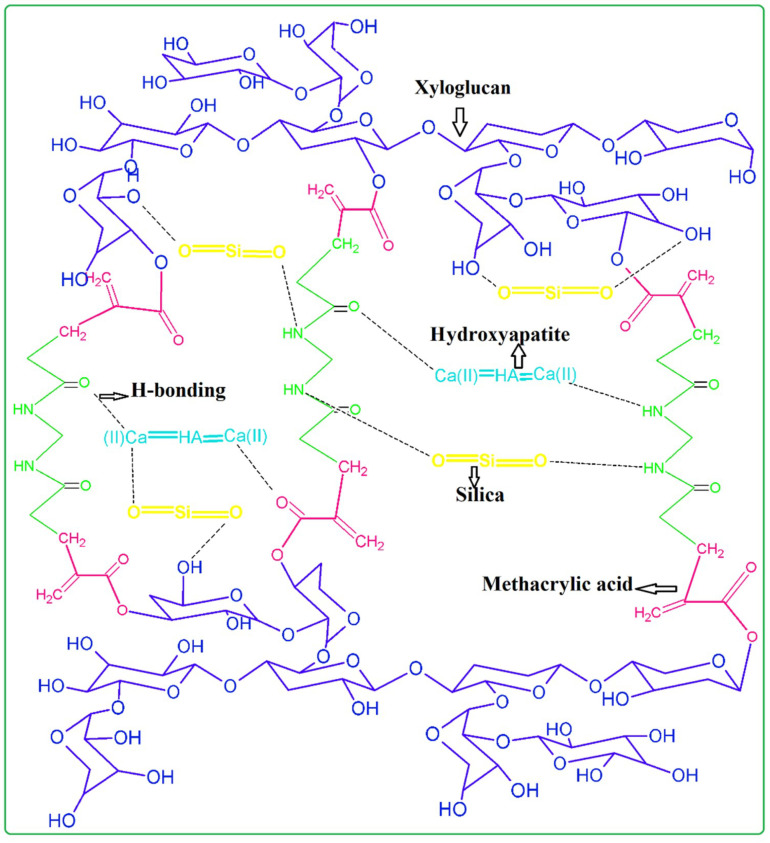
Proposed chemical reaction scheme of polymeric bioactive scaffolds fabrication of n-HAp and n-SiO_2_ doped in a grafted biopolymeric matrix of XG.

Polymeric bioactive scaffolds were produced using the freeze-drying method. The doped n-HAp and n-SiO_2_ were accompanied through a free-radical polymerization process in a grafted polymer matrix of XG and MAAc. Figure 2 indicates the relationships between the materials and possible chemical reactions.

### 4.1. FT-IR

**Figure 3:**
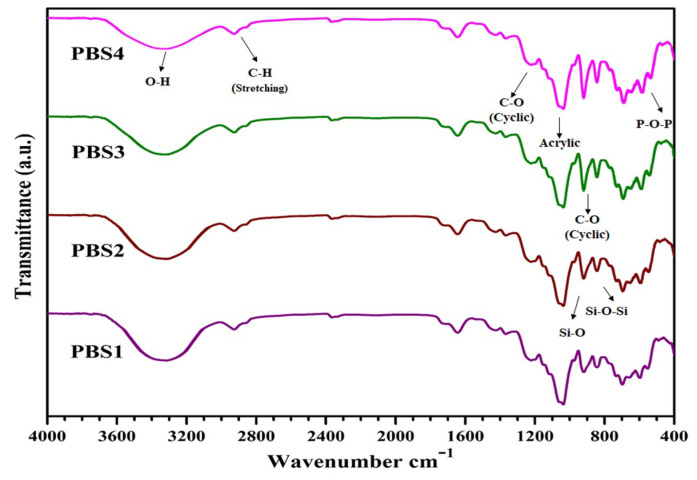
Typical spectral Fourier transform infrared spectroscopy (FTIR) profiles present peaks for different functional groups and their various modes of vibrations for all samples of polymeric bioactive scaffolds.

Figure 2 shows the proposed chemical interactions among XG, MAAc, N, N-MBA, n-HAp and n-SiO_2_. Stretching vibrations at 1220 cm^−1^,1033 cm^−1^ and a weak peak at 906 cm^−1^ are due to C–O cyclic, acyclic and pyranose, respectively and it might be due to formation of the covalent bond between XG and MAAc as shown in Figure 2 [24]. FTIR spectra represent specific functional groups in polymeric bioactive scaffolds. Bands from 3600 to 3100 cm^−1^ (Figure 3) are due to hydroxyl stretching vibrations. The band absorption at 2950–2850 cm^−1^ shows the aliphatic C–H stretching vibration. The peak features at 1093 cm^−1^ are due to triply degenerated P–O stretching components, while peaks at 603 and 569 cm^−1^ are attributed to O–P–O bending mode. Hence, FTIR spectra present the characteristic bands in regions 560–600 cm^−1^ and 1000–1100 cm^−1^ are assigned to calcium phosphate moiety of n-HAp [25]. Furthermore, the absorption band at 630 cm^−1^ confirms the presence of n-HAp in all the fabricated polymeric bioactive scaffolds [26,27]. The 947 cm^−1^ band defines the formation of Si–O hydrogen-bonding with XG hydroxyl groups. Rising peak intensity from 947 to 921 cm^−1^ was observed as n-SiO_2_ increased. The H-bond formation between the oxygen atom of n-SiO_2_ and hydrogen atoms of the hydroxyl group of XG is presented in Figure 3. The characteristic peak is shown by n-SiO_2_ from 600 to 800 cm^−1^ presenting the stretching vibration of Si–O–Si for polymeric bioactive scaffolds [28]. Analysis of the FT-IR spectral profile confirms the successful synthesis of nanocomposite for the development of bioactive scaffolds.

### 4.2. Mechanical Testing

**Figure 4:**
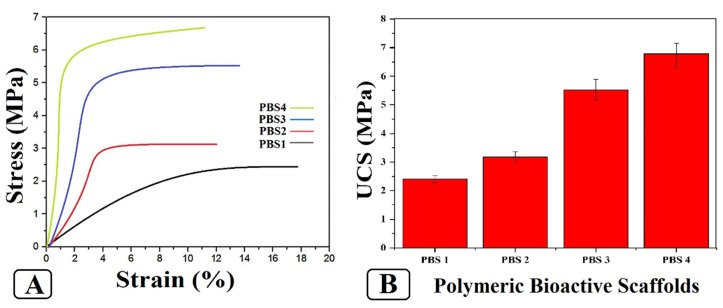
Different mechanical behavior of polymeric bioactive scaffolds. (**A**) Strain-stress curve of polymeric bioactive scaffolds and (**B**) Ultimate compression strength data curves of polymeric bioactive scaffolds.

Figure 4 illustrates the compression strength of bioactive scaffolds samples in our study. It is highly challenging but important to maintain the mechanical strength of our scaffolds during cell growth and proliferation. It not only physically safeguards the cells but also provides compatible mechanical strength to mimic in vivo environments that have stronger influences over cell proliferation and differentiation. The ceramic phase (n-HAp and n-SiO_2_) has an extraordinary role to increase biomechanical features of several polymeric materials [29,30]. The incorporation of n-SiO_2_ increased the compression strength of the polymeric bioactive scaffolds (UCS of PBS1 = 2.4 MPa, PBS2 = 3.2 MPa, PBS3 = 5.7 MPa, PBS4 = 6.9 MPa). The sample PBS3 has better efficacy to bear the load with suitable porosity levels that eventually increased the mechanical properties of the fabricated polymeric bioactive scaffold. All polymers have different chemical structures that affect the grain boundary of the matrix. The properties of ceramic material (n-HAp and n-SiO_2_) is a function of pore size, porosity and grain boundary that is decreased with decreasing in grain size.

### 4.3. Swelling Analysis

**Figure 5:**
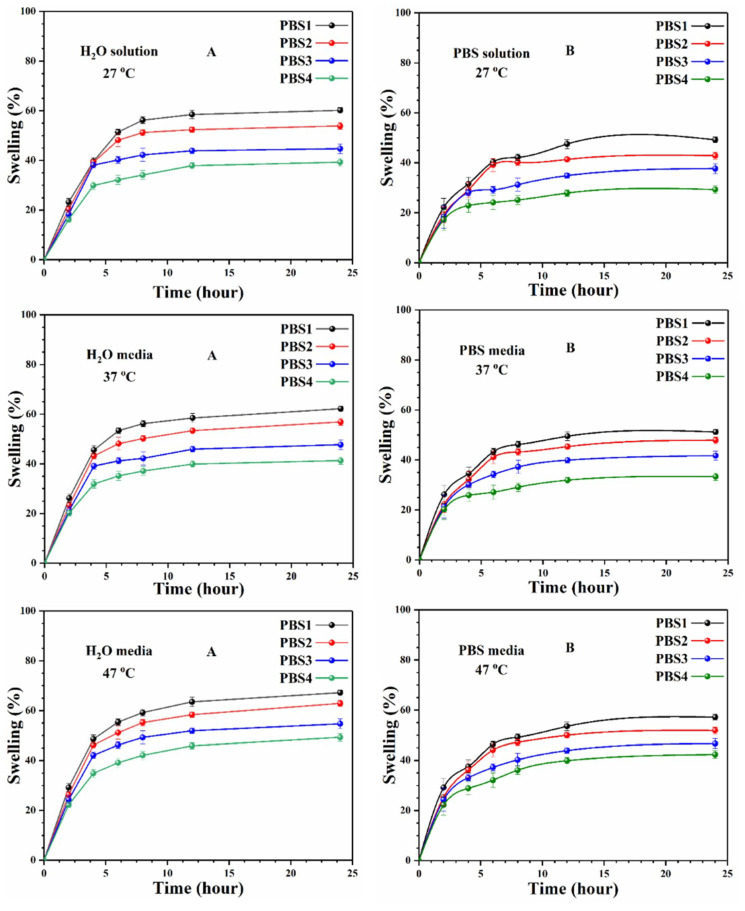
The swelling analysis was carried out at different temperatures (27, 37 and 47 °C) and found that not only is the quantity of silica responsive to swelling changes but the increasing temperature is also responsible for increased swelling. (**A**) In deionized-water (**B**) In phosphorous buffer saline solution.

Figure 5 discusses the swelling analysis of polymeric bioactive scaffolds in deionized-water and phosphorous buffer saline solution. Identical amounts of scaffolds were immersed in deionized water and PBS solution. The polymer bioactive scaffolds are found to have diverse swelling patterns in different media at different temperatures, because of different n-SiO_2_ concentrations. Osmotic pressure may increase or decrease because of the hydrogen bonding (H- bonding) among the scaffold and media at varying temperatures. The swelling rate decreased and osmotic forces are balanced at equilibrium after increasing the time. The extraordinary difference in water absorption is not observed with time until it has reached a plateau [31]. Initially, more water molecules interacted with porous scaffolds and decreased when the equilibrium point is near. Sample (PBS1) with the maximum amount of n-SiO_2_ has maximum swelling in water media at 47 °C. Whereas, sample (PBS4) with a little amount of silica at 27 °C has the least swelling. It is obvious (Figure 5) that swelling has an inverse relationship to the ceramic quantity within the polymeric network. Due to the relatively hydrophobic nature of ceramic materials in XG-graft-MAAc and ceramic materials, the different swelling behaviors of these bioactive scaffolds can react as a crosslinker. As a consequence, by increasing ceramic quantities into a polymeric network, which generates additional crosslink points in polymer networking and decreases elasticity which decreases swelling behavior.

### 4.4. SEM-EDX

**Figure 6:**
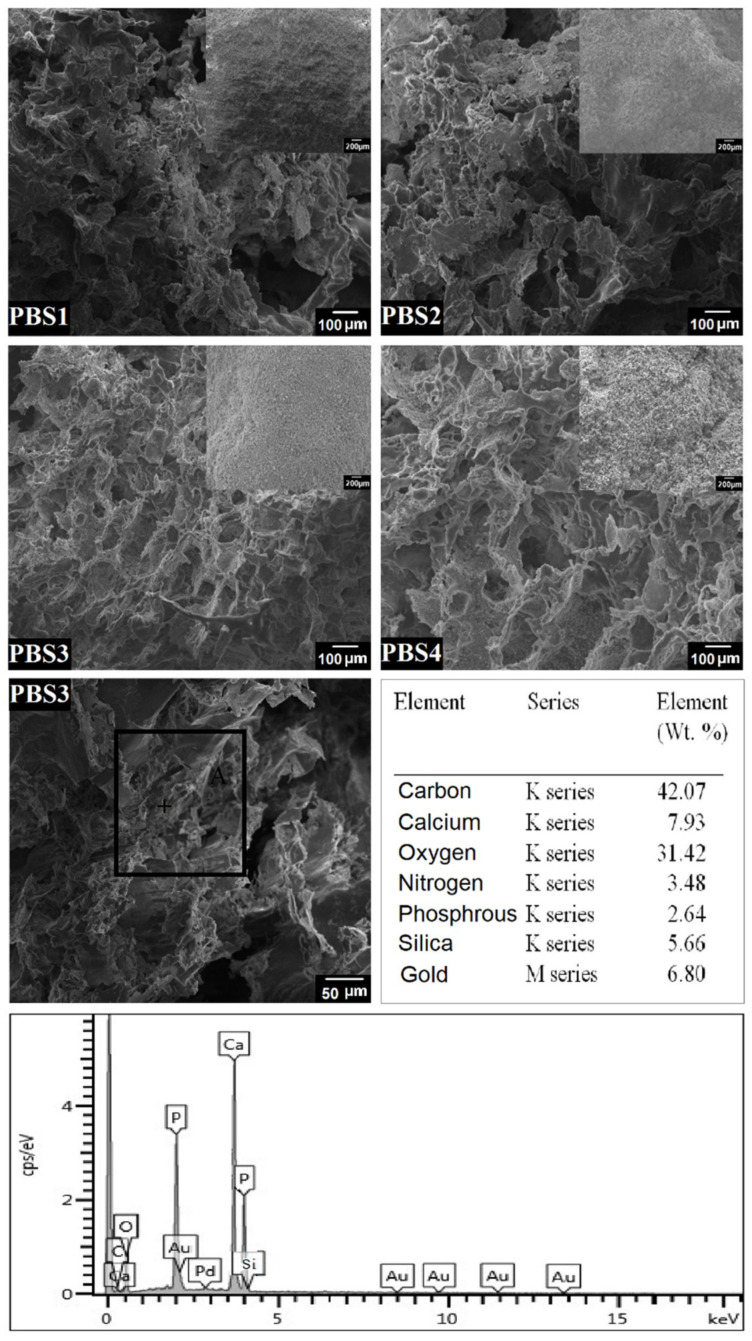
Surface morphology and elemental analysis of all polymeric bioactive scaffold samples.

The surface morphology of the bioactive polymeric scaffolds was examined using SEM as shown in Figure 6. The various magnifications exhibit rough and porous topography of polymeric bioactive scaffolds (evident from the XRD results), which remarkably encourage cell adhesion and proliferation on the scaffold [32,33]. At higher magnification, however, the SEM images show a specific porous morphology which also supports cell infiltration, adhesion and extracellular matrix secretion. The bioactive polymeric scaffolds show different pore sizes distributed uniformly through the samples. The average pore size of PBS3 is 80–140 μm, which is ideal for osteointegration and 50–200 μm is optimal for cell attachment, proliferation and osteoblast cells. The PBS3 sample has a uniform distribution of pore size in the structure and is ideal for significant cell proliferation demonstrating excellent cell growth capability. [34]. During the SEM analysis, n-SiO_2_ was found to have imparted its role in porosity, as the increasing quantity of n-SiO_2_ caused increased pores and porosity. The PBS3 sample was chosen because of the uniform pore size used for chemical composition analysis with EDX [33]. EDX study reveals the different amounts of elements present in sample PBS3. Results show the presence in the porous bioactive scaffold of carbon (C), oxygen (O), silica (Si), calcium (Ca) and phosphorus (P) elements (Figure 6), which help grafted-XG in manufactured bioactive polymer scaffolds.

### 4.5. In-Vitro Study

The polymeric bioactive scaffolds samples were selected for in-vitro analysis due to their optimum characteristics.

#### 4.5.1. Antibacterial Activities

**Figure 7:**
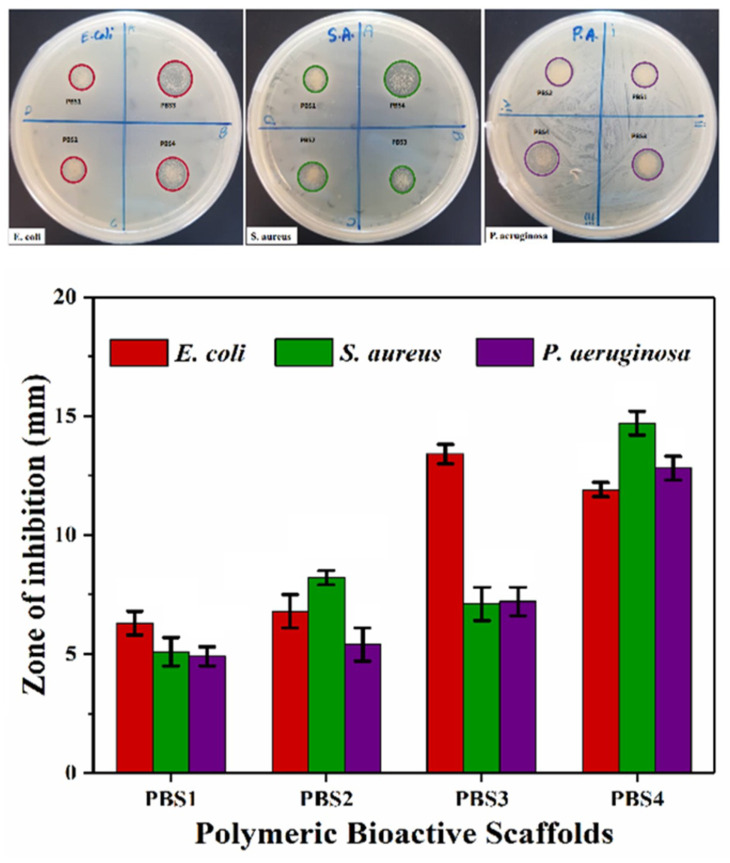
Antibacterial behavior of all bioactive polymeric scaffolds against different bacterial species (grams-positive and grams-negative). These bioactive scaffolds have exhibited numerous antibacterial activities which can be seen through inhibition zones.

Antibacterial activities of scaffolds were performed via agar disc-diffusion assay against various sever pathogens (*E. coli*, *S. aureus* and *P. aeruginosa*) and zone of inhibition was measured as seen in Figure 7 [35]. Antibacterial activity of scaffolds is due to the penetration of silica and hydroxyapatite nano-particles into bacteria to interact with the cellular protein. The denatured proteins were caused by silica and hydroxyapatite nanoparticles and by the accretion of external fluids to give silica/hydroxyapatite scaffolds better antibacterial activities. The charged bacterial surface membrane (phospholipids and lipopolysaccharides) interacts with several functional groups of bioactive polymeric scaffolds [36,37]. These active sites of bioactive polymeric scaffolds allowed their changes into the bacterial membrane to obstruct bacterial activity. Bacterial growth is overdue due to the interaction between the polymeric portion of scaffold and bacterial DNA. Molecular alteration in DNA because of polymeric and SiO_2_/HAp nanoparticles of bioactive polymeric scaffolds that caused little to no bacterial growth. All samples of polymeric bioactive scaffolds, therefore, exhibited antibacterial activity due to components of polymeric bioactive scaffolds and also increasing quantities of silica nanoparticles [38].

#### 4.5.2. Cytotoxicity

**Figure 8:**
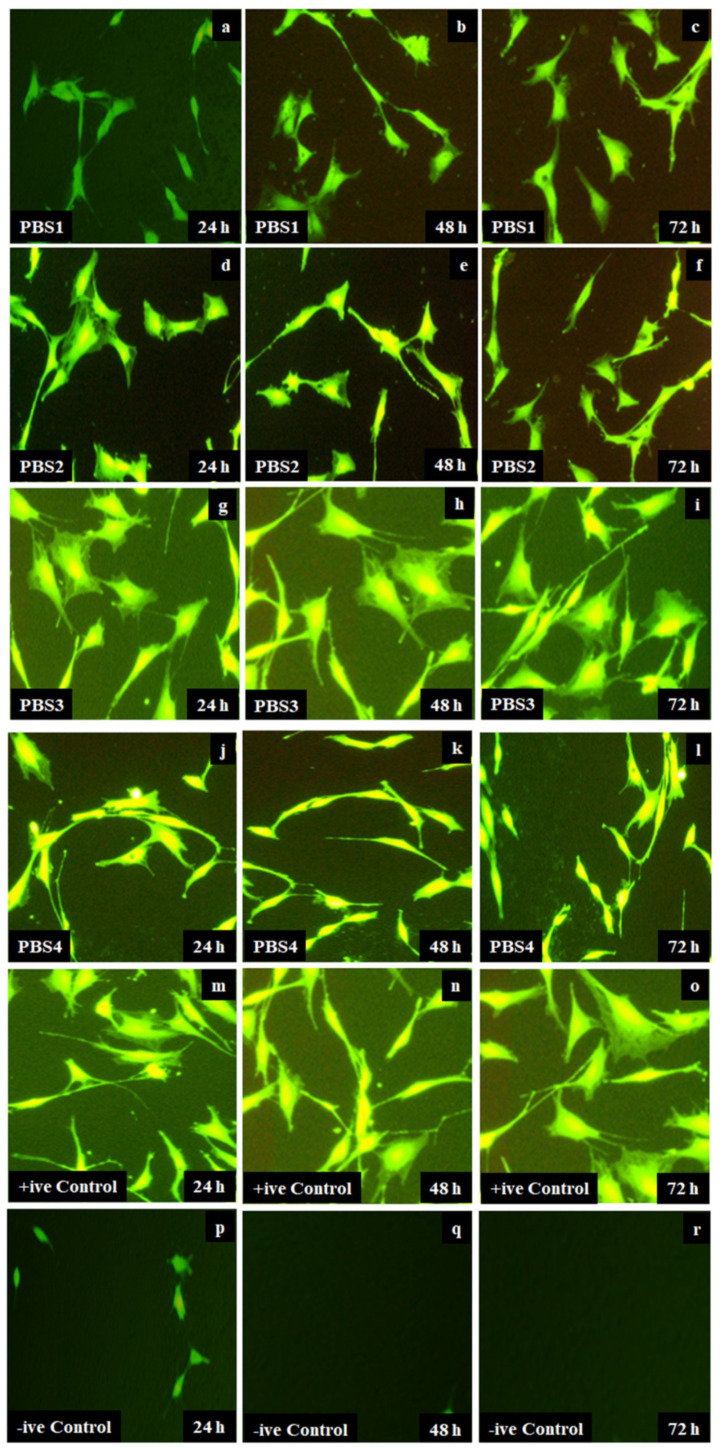
The optical images cell morphology that is taken by an optical microscope of all polymeric bioactive scaffolds (PBSs) samples PBS-1 (**a**–**c**), PBS2 (**d**–**f**), PBS3 (**g**–**i**), PBS4 (**j**–**l**), +ve control (**m**–**o**) and –ve control (**p**–**r**) at different time interval 24, 48 and 72 h.

All scaffolds were incubated into culture medium at 37 °C for 24, 48 and 72 h. Culture media contained 90% DMEM glucose followed by 2 mmol L^−1^ glutamine, 10% FBS and 1% penicillin. Attached cells demonstrate ideal MC3T3-E1 cell line morphology for all samples of polymeric bioactive scaffolds, where cells are cylindrical with proper cell bodies. Cells attached to the well-bottom are comparatively less retained morphology as compared to control [33,39]. Figure 8, on the contrary, detects a particular pattern. PBS3 is found to have become more supportive of cells as it has added a large number of cells to the bottom of the well plate without any deformation of cell morphology compared to +ve-control. Whereas PBS1 has the least growth in cells compared to PBS3 because of an increasing amount of the n-SiO_2_. The increasing amount of silica makes scaffolds more biocompatible and increases the growth of *MC3T3-E1* over time. In comparison, the negative control depicted smaller or no cells growth. It is therefore evident from the results that grafted XG-based biomaterials strengthened osteoblast cell attachment by increasing the n-SiO_2_ content.

#### 4.5.3. Neutral Red Assay

**Figure 9:**
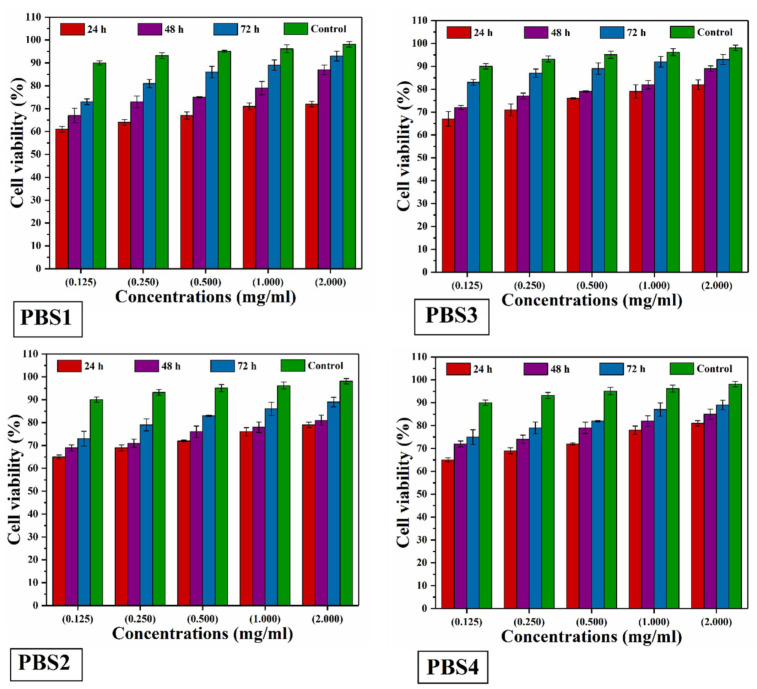
The cell viability of all polymeric bioactive scaffold samples was investigated using their extracts (0.125 to 2.00 mg mL^−1^). These five different concentrations have incubated using pre-osteoblast (*MC3T3-E1*) cells at different time intervals (24, 48 and 72 h) at 37 °C.

Cytotoxicity assay was carried out using the pre-osteoblast mouse (MC3T3-E1) cells by rising against all scaffolds. Cell viability was evaluated for each concentration of all samples and recorded at different time intervals using Neutral red uptake assay by incubation (24, 48 and 72 h) [31,33]. As the amount of n-SiO_2_ increases, polymeric bioactive scaffolds show greater cell viability and non-toxicity against pre-osteoblast mouse (*MC3T3-E1*) cells (Figure 9). PBS3 has no or less toxic cell viability that promotes pre-osteoblast (MC3T3-E1) cell differentiation to help the bone formation and plays an important role in tissue engineering for bone regeneration.

#### 4.5.4. SEM Analysis of Cell Culture

**Figure 10:**
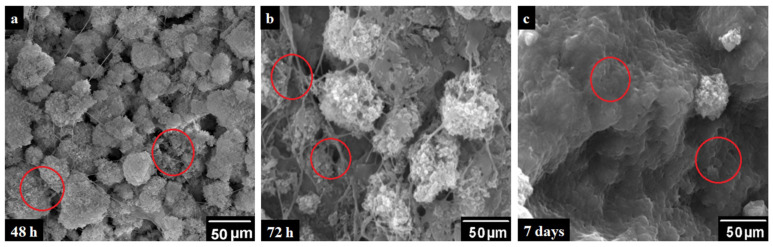
Scanning electron microscopy (SEM) micrographs of cell growth over PBS3 at (**a**) 48 h, (**b**) 72 h and (**c**) 7 days, at 100 μm and upper right at 200 μm respectively and the red circle shows the cell growth area.

The PBS3 sample was selected for SEM analysis because of optimal swelling, mechanical, porosity, pore size, antibacterial and biocompatibility. Adherence of MC3T3-E1 pre-osteoblast cells to PBS3 was analyzed using SEM (Figure 8) and presented via micrographs (A) PBS3, (B) Positive control (C) after 48 h, 72 h and 7 days of cultivation, respectively [31,33]. After specific incubation time (48 h, 72 h and 7 days), the pre-osteoblast MC3T3-E1 cells responded to PBS3 by exhibiting bioactive and unique cell attachment, which spread all over the surface (Figure 10). Hence, the PBS3 was found to good in cell adherence and proliferation [33,39].

## 5. Conclusions

Polysaccharides are prominent polymeric biomaterials for many biomedical applications. We have used XG to fabricate polymeric bioactive scaffolds for bone tissue engineering applications. The different amounts of n-SiO_2_ have different porosity, pore size, swelling and biomechanical behavior of these polymeric bioactive scaffolds for support and growth bones. The ultimate compression strength of PBS3 is ~5.7 MPa, which is closer to cancellous bone strength. PBS3 polymeric bioactive scaffold exhibited adequate porosity (86.5% ± 1%) with a pore size (105 ± 2 µm) that is essential for osteogenesis. PBS3 was found to be antibacterial against several infections causing pathogens. PBS3 is a more biologically active scaffold that encouraged osteoblast cell line growth with adequate cell viability and cell growth. The overall outcome has verified that polymeric bioactive scaffolds are potential biomaterial for bone tissue engineering.

## 6. Limitations

The increasing amount of ceramic contents (n-SiO_2_) changes behavior from polymeric to amorphous that also changed the physicochemical properties of the scaffolds. Further increasing amount of n-SiO_2_ causes a bigger pore size that fails mechanical properties that may not support these scaffolds mechanically. Since we are investigating other possible material to increase more mechanical properties by enhancing physicochemical and biological properties.

## Figures and Tables

**Table 1:** Percentage of porosity, pore size and n-SiO_2_ amount.

Samples	n-SiO_2_ (g)	Pore Size (µm)	Porosity (%)	Compression Strength (MPa)
PBS1	0.25	97 ± 2.43	65.5 ± 1.31	2.49 ± 1
PBS2	0.50	107 ± 3.42	75.2 ± 3.41	3.34 ± 2
PBS3	0.75	132 ± 5.06	84.5 ± 6.21	5.61 ± 1
PBS4	1.00	173 ± 8.12	91.5 ± 4.19	6.94 ± 1
